# Long-term exposure to low-level arsenic in drinking water is associated with cause-specific mortality and hospitalization in the Mt. Amiata area (Tuscany, Italy)

**DOI:** 10.1186/s12889-022-14818-x

**Published:** 2023-01-10

**Authors:** Daniela Nuvolone, Giorgia Stoppa, Davide Petri, Fabio Voller

**Affiliations:** 1 Unit of Epidemiology, Regional Health Agency of Tuscany, Via Pietro Dazzi 1, 50124 Florence, Italy; 2 Unit of Biostatistics, Epidemiology and Public Health, DCTVPH, University of Padua, 35131 Padua, Italy; 3 Department of Clinical and Experimental Medicine, University of Pisa, 56126 Pisa, Italy

**Keywords:** Arsenic, Drinking water, Residential cohort study, Volcanic areas, Mortality, Hospitalization

## Abstract

**Background:**

Arsenic in drinking water is a global public health concern. This study aims to investigate the association between chronic low-level exposure to arsenic in drinking water and health outcomes in the volcanic area of Mt. Amiata in Italy, using a residential cohort study design.

**Methods:**

Chronic exposure to arsenic in drinking water was evaluated using monitoring data collected by the water supplier. A time-weighted average arsenic exposure was estimated for the period 2005–2010. The population-based cohort included people living in five municipalities in the Mt. Amiata area between 01/01/1998 and 31/12/2019. Residence addresses were georeferenced and each subject was matched with arsenic exposure and socio-economic status. Mortality and hospital discharge data were selected from administrative health databases. Cox proportional hazard models were used to test the associations between arsenic exposure and outcomes, with age as the temporal axis and adjusting for gender, socio-economic status and calendar period.

**Results:**

The residential cohort was composed of 30,910 subjects for a total of 407,213 person-years. Analyses reported risk increases associated with exposure to arsenic concentrations in drinking water > 10 µg/l for non-accidental mortality (HR = 1.07 95%CI:1.01–1.13) and malignant neoplasms in women (HR = 1.14 95%CI:0.97–1.35). Long-term exposure to arsenic concentrations > 10 µg/l resulted positively associated with several hospitalization outcomes: non-accidental causes (HR = 1.06 95%CI:1.03–1.09), malignant neoplasms (HR = 1.10 95%CI:1.02–1.19), lung cancer (HR = 1.85 95%CI:1.14–3.02) and breast cancer (HR = 1.23 95%CI:0.99–1.51), endocrine disorders (HR = 1.13 95%CI:1.02–1.26), cardiovascular (HR = 1.12 95%CI:1.06–1.18) and respiratory diseases (HR = 1.10 95%CI:1.03–1.18). Some risk excesses were also observed for an exposure to arsenic levels below the regulatory standard, with evidence of exposure-related trends.

**Conclusions:**

Our population-based cohort study in the volcanic area of Mt. Amiata showed that chronic exposure to arsenic concentrations in drinking water above the current regulatory limit was associated with a plurality of outcomes, in terms of both mortality and hospitalization. Moreover, some signs of associations emerge even at very low levels of exposure, ​​below the current regulatory limit, highlighting the need to monitor arsenic concentrations continuously and implement policies to reduce concentrations in the environment as far as possible.

## Background

Arsenic (As) is a ubiquitous element in the environment, and it occurs in both organic and inorganic forms. Human exposure to arsenic occurs mainly through the ingestion of contaminated food and water and, to a lesser extent, by inhalation and dermal contact. Organic compounds, which are less harmful than inorganic compounds, are most abundant in food, while inorganic forms are mainly found in water, including that intended for human consumption [1].

The health effects of exposure to high levels of arsenic in drinking water are well documented in studies of populations exposed in endemic areas, such as some Asian countries (Bangladesh, Taiwan, Vietnam, India), Argentina, Chile, and several areas of the United States (Arizona, California and Nevada) [2–10]. Based on sufficient evidence of carcinogenicity in humans and limited evidence of carcinogenicity in animals, arsenic and inorganic arsenic compounds have been classified by the International Agency for Research on Cancer (IARC) in Group 1 (carcinogenic to humans), due to an increased risk of lung, bladder and skin cancers [11]. There is also limited evidence of carcinogenesis reported for cancers of the liver, kidney and prostate, and there are reports of the effects of exposure to high levels of arsenic in drinking water on non-oncological outcomes, such as cardiovascular and respiratory diseases, diabetes, cognitive development and adverse pregnancy outcomes [3, 9, 10, 12].

At lower doses, the effects of prolonged exposure to arsenic have not yet been fully characterized [12–15]. Adverse effects have been reported on various systems, such as foetal development, glucose metabolism, skin pigmentation and peripheral vascular diseases have been reported [9, 16–18]. However, the evidence available is insufficient to identify a dose–response relationship or a threshold below which effects on health are excluded [13, 16, 18].

The widespread presence of arsenic in groundwater is a common feature of several areas of Italy. Mt. Amiata is the second tallest volcano in Italy located in southern Tuscany (Central Italy), and covers an area of about 80 km^2^. The volcanic rock is highly permeable due to fractures and houses an important potable phreatic aquifer. Due to the good hydraulic qualities and the relatively large volume of the volcanic rock, this aquifer represents the main water resource for a large part of southern Tuscany [19]. The Mt. Amiata area has been the subject of intensive geological and geophysical investigations, mainly for geothermal resources [20–22]. There are two geothermal reservoirs in the area and these have been exploited industrially for electricity production [23]. Epidemiological studies have also been conducted to evaluate the effects of exposure to hydrogen sulphide and other emissions from geothermal power plants on population health [24–27]. Moreover, in the past Mt. Amiata was also known as a world-class mercury (Hg) mining district, where the metal occurred as cinnabar and had been exploited since Etruscan times [28–30]. Production increased sharply in the second half of the 1800s [31, 32], as indicated by Ferrara et al. [33], who reported that from 1860 to 1980, when all Hg mines were permanently closed, more than 100,000 t of Hg were produced in the Mt. Amiata mining district, and it was ranked as the 4th largest producing district in the world [34].

In 1998 according to new evidence from the literature [35], the legal limit for arsenic concentrations in drinking water was lowered from 50 µg/l to 10 µg/l (European Drinking Water Directive 98/83/EC, implemented in Italy through Legislative Decree 31/2001). For several years after the entry into force of the new regulatory standard, various municipalities in Italy, including in the Mt. Amiata area, were granted derogation. In 2010, the European Union resolved not to grant any further derogations and since then actions have been taken and structural works implemented to restore water quality. However, for decades populations in the Amiata area have been exposed to concentrations of arsenic in drinking water higher than the current regulatory limit.

The main objective of this study is to evaluate the long-term effects of chronic exposure to arsenic in drinking water in the area of Mt. Amiata, using a residential cohort study design.

## Materials and methods

### Definition of the cohort

The population-based cohort comprised all residents in the municipalities of Piancastagnaio, Abbadia San Salvatore, Arcidosso, Santa Fiora and Castel del Piano from 1 January 1998, and all those registered up to 31 December 2019. This is an open and dynamic cohort and all demographic movements were considered, such as births, immigration entries, emigration exits, deaths and changes of home address. The demographic archives transmitted by the registry offices of each municipality were subjected to quality control procedures, with the removal of duplicated entries, the correction of errors in the sequence of dates, and exclusion of subjects registered on the Registry of Italians Residing Abroad (AIRE). Geographical coordinates were assigned to each address and all subjects were included in a geographic information system (GIS). The georeferencing results were subjected to data quality analysis by overlapping with orthophotos and regional technical maps, to verify the degree of completeness and the level of precision of the geocoding process. Each subject of the study cohort was also assigned a value on the socio-economic deprivation index for the census tract where they live. This index is based on information on education, unemployment, number of rented dwellings, number of single-parent families with dependent children and housing density [36].

### Arsenic concentrations in drinking water

Since 2005, the integrated water distribution system of the Mt. Amiata area has been operated by Acquedotto del Fiora Spa. According to the requirements established by Italian Legislative Decree 31/2001, arsenic is monitored at specific sampling points and a number of water samples are collected annually, proportional to population size and equally distributed in time and location. The analytical determination of arsenic is carried out by means of atomic absorption spectrometry and inductively coupled plasma mass spectrometry. Monitoring campaigns are carried out by the water supplier and the Local Health Authority. Acquedotto del Fiora provided data on arsenic concentration in tap water samples from 2005 to 2010. Data collected by the Local Health Authority have been available in paper format since 1988, but before 2001, when the new limit of 10 μg/l came into force, the arsenic measurements were fairly inaccurate and are not comparable with those taken in more recent years. Consequently, in our study we assumed that before 2005 the arsenic concentrations were stable in the study period based on the widely known levels of arsenic in groundwater, due to natural underlying geological processes and the absence of any mitigation action prior to 2010.

**Fig. 1:**
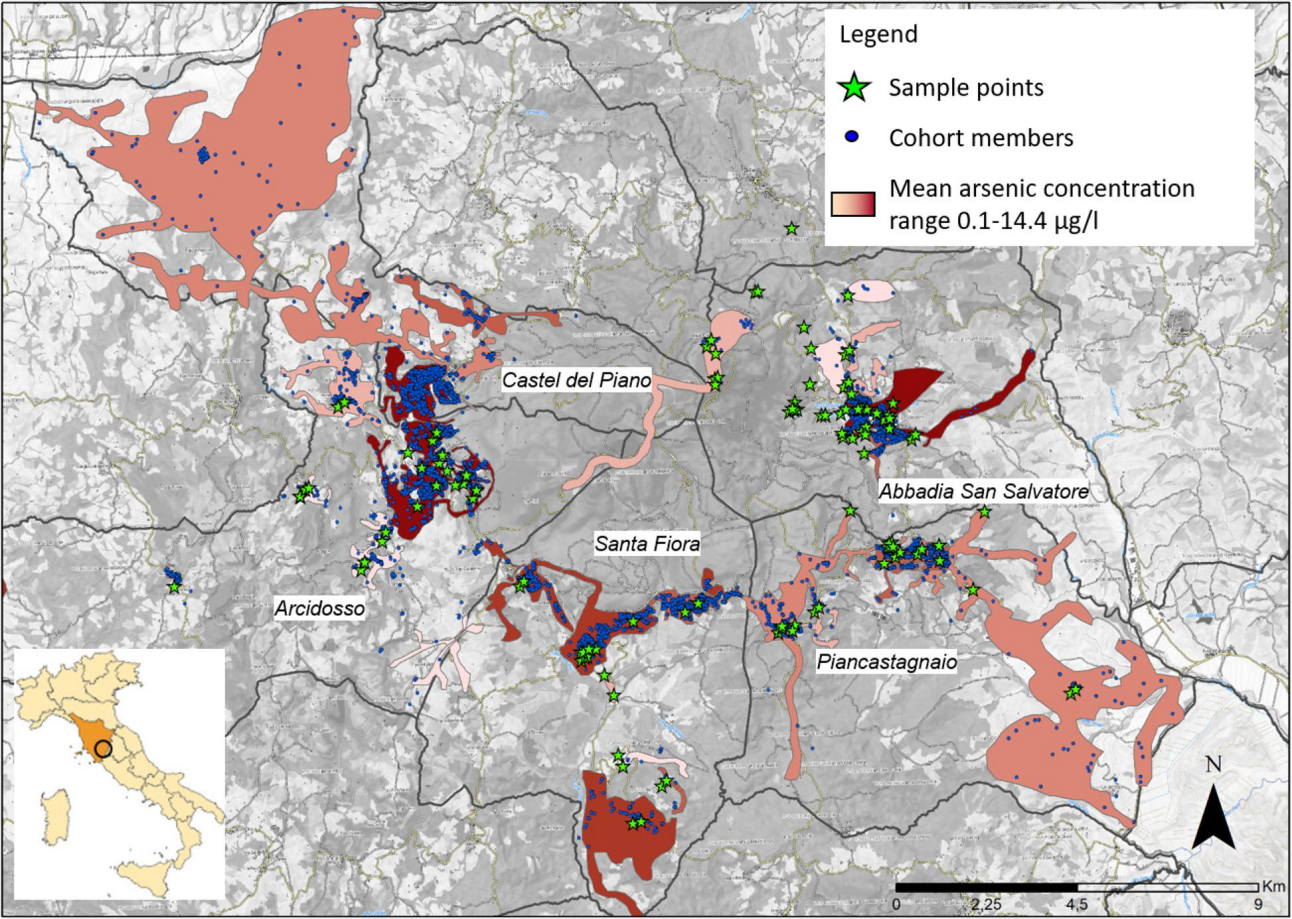
Map of the supply units for the five municipalities under study

In addition to tap water data, the geographic coordinates of sampling points were also integrated into the geographic information system. Acquedotto del Fiora also divided the municipal territory into several supply units which deliver water directly to households. These supply units are portions of the territory considered to be homogeneous in terms of the type and quality of tap water distributed to households. They were identified by taking into account the quality of springs and groundwater, the characteristics of the water distribution system and the dilution procedures carried out by the water supplier. Figure 1 shows the map of the supply units for the five municipalities under study, with indication of the water sampling points (green stars) and the homes of the cohort members (blue dots).

Long-term exposure to arsenic concentrations in tap water was analysed for each indivudual. Each set of home coordinates was overlapped with the water supply unit using the GIS spatial join function, applying the average arsenic concentration in the period 2005–2010. For homes that did not fall within the supply units (rural locations) the subjects were assigned the arsenic concentrations of the nearest water utility, using the “point-to-point” spatial join.

To estimate the long-term exposure to arsenic in drinking water, we calculated a time-weighted average exposure (TWA) for each cohort member, adding for each home the arsenic levels multiplied by the time they lived at this address, divided by the total observation time, according to the following formula:

$$TWA\;=\frac{{\sum\limits_i}{As}_{\mathrm i}\;\times\;D_i}{\sum\limits_{i} D_i}$$

where As_i_ (in µg/l) is the average 2005–2010 concentration of arsenic at the i-th address and D_i_ is the time lived at the i-th address.

### Follow-up procedures and health data

For each subject of the cohort, we performed the vital status follow-up from 01/01/1998 to 31/12/2019, using the municipal registry database. For subjects who died in the period 1998–2016, the cause of death was attributed using data from the Tuscany Regional Mortality Register (RMR), active since 1987, which records the deaths of residents of the Tuscany region, occurring both inside and outside the region. The procedure for identifying the cause of death was anonymous using a code applied by the Tuscany region for the purpose of protection of privacy.

Hospital admissions were analysed using data from the Hospital Discharge Records (HDR) of the Tuscany region, related to ordinary and day-hospital admissions, occurring inside and outside the region in the period 1998–2019. Considering incident cases only, a subject hospitalized several times for the same disease was counted only once, using the date of the first hospital admission. Furthermore, analyses only considered the primary diagnosis at discharge.

### Statistical analysis

Each member of the cohort contributed to the calculation of person-years at risk starting from 1 January 1998 if living in the municipality on this date or starting from the date of their registration on the municipal registry if entering the municipality after 01/01/1998, up to the date of death, emigration or end of follow-up (31 December 2016 for mortality analyses and 31 December 2019 for hospitalization analyses).

The associations between long-term exposure to arsenic in drinking water and mortality/morbidity outcomes were evaluated using Cox proportional hazard models, to estimate hazard ratios (HR) and 95% confidence intervals [37]. The Cox models used age as the time scale and the confounding effects of gender, socio-economic deprivation indicator and calendar periods were taken into account. The assumption of the proportionality of the risks was tested using the Mantel-Cox test and, if violated, stratified Cox models were applied.

Two distinct approaches were used to assess exposure. In the first approach, the TWA variable was introduced to the models as a two-level categorical variable, comparing concentrations greater than 10 µg/l with concentrations equal to or lower than 10 µg/l. In the second approach, to assess the risks associated with arsenic concentrations below the regulatory limit of 10 µg/l, the TWA variable was introduced to the models as a three-level categorical variable, using concentrations ≤ 5 µg/ l as the reference group and two increasing exposure groups, 5–10 µg/l and ≥ 10 µg/l. *P*-values were calculated in the three-level models to identify trends.

## Results

The cohort included 30,910 residents, 14,970 men and 15,940 women, for a total of 407,213 person-years. The main socio-demographic characteristics of the cohort are shown in Table 1.

**Table 1:** Socio-demographic characteristics of the cohort

	Men		Women		Total	
	n	%	n	%	n	%
	14,970		15,940		30,910	
Person-years	195,794		211,419		407,213	
Age at the baseline						
Mean (± SD)	38.9 (± 24.2)		42.8 (± 25.4)		40.9 (± 24.9)	
Age groups						
< = 35 years	6941	46.4	6495	40.8	13,436	43.5
36–64 years	5323	35.6	5656	35.5	10,979	35.5
> = 65 years	2706	18.0	3789	23.7	6495	21.0
Municipality						
Abbadia San Salvatore	4392	29.3	4829	30.3	9221	29.8
Arcidosso	2867	19.2	2908	18.2	5775	18.7
Castel del Piano	2899	19.4	3130	19.6	6029	19.5
Piancastagnaio	2671	17.8	2835	17.8	5506	17.8
Santa Fiora	2141	14.3	2238	14.1	4379	14.2
Years of residence						
> = 15 years	8801	58.8	9612	60.3	18,413	59.6
Socio-economic status						
High	8313	55.5	8635	54.1	16,948	54.8
Medium	3114	20.8	3492	21.9	6060	21.4
Low	3475	23.2	3743	23.5	7218	23.4
Missing	68	0.5	68	0.4	136	0.4

Abbadia San Salvatore was the most populous municipality and made up 29.8% of the study cohort. About 60% of the population had lived in the study area for more than 15 years, and 54.8% had a high socio-economic status. A very high proportion of georeferenced addresses (98%) and good accuracy were achieved, also thanks to the manual recovery of non-geocoded addresses using automated procedures.

Table 2 shows the main characteristics of the drinking water supply units, reporting the number of water sampling points, the number of people served and the average arsenic concentrations in the period 2005–2010.

**Table 2:** Characteristics of drinking water supply units

Municipality	Supply unit code	N. of water sample points	N. of people served	Mean As concentrations 2005–2010 (μg/l)
ABBADIA SAN SALVATORE	ASSDQ1	38	644	3.39
	ASSDQ3	27	29	3.05
	ASSDQ4	6	2682	7.80
	ASSDQ5	66	1949	11.75
	ASSDQ6	104	7381	14.37
	ASSDQ7	17	47	1.80
	ASSDQ8	24	125	1.38
	ASSDQ9	60	708	9.19
ARCIDOSSO	ARCDQ1	174	5024	10.10
	ARCDQ2	32	444	0.66
	ARCDQ3	39	838	2.36
	ARCDQ4	75	1309	9.70
	ARCDQ5	11	157	1.56
	ARCDQ6	13	365	4.23
	ARCDQ7	5	29	8.50
CASTEL DEL PIANO	CPIDQ1	50	6423	10.74
	CPIDQ2	30	357	6.07
	CPIDQ3	11	1174	5.55
	CPIDQ4	16	320	6.19
	CPIDQ6	4	120	3.70
	CPIDQ7	2	298	6.15
PIANCASTAGNAIO	PCADQ1	166	2818	5.70
	PCADQ2	24	335	3.64
	PCADQ3	34	5141	6.84
	PCADQ4	62	515	5.99
SANTA FIORA	SFIDQ1	51	3934	9.00
	SFIDQ2	14	1447	6.78
	SFIDQ4	2	990	9.00
	SFIDQ7	13	13	6.25
	SFIDQ8	13	35	0.89

Table 2 shows the spatial variability of arsenic levels, with the highest value recorded in Unit 6 of Abbadia San Salvatore ASSDQ6 (mean arsenic concentration = 14.37 µg/l). Conversely, the lowest value is observed in Unit 2 of Arcidosso ARCDQ2 (mean arsenic concentration = 0.66 µg/l).

Cohort members were exposed to a time-weighted average (TWA) concentration of arsenic equal to 9.2 µg/l (range: 0.7–14.4 µg/l). Table 3 shows the distribution of the cohort members by gender, age and socio-economic status for the two classes of exposure to arsenic in drinking water.

**Table 3:** Socio-demographic characteristics of the cohort members by arsenic exposure

	**TWA**^**a**^** < 10 µg/l**	**TWA ≥ 10 µg/l**	*p*-value**
Total	17,139	13,730	
Gender			
Men	8400 (56.1)	6552 (43.8)	
Women	8739 (54.9)	7178 (45.1)	0.120
Mean age (years)	40.1	41.9	0.098
Socio-economic status			
High	9746 (57.6)	7183 (42.4)	
Medium	4594 (69.5)	2012 (30.5)	
Low	2687 (37.3)	4512 (62.7)	< 0.001

^a^TWA: time-weighted average arsenic exposure in the period 2005–2010

^**^ *p*-value from χ^2^ and T-test

Forty-four percent of the cohort members were exposed to arsenic concentrations higher than the current regulatory limit. There were no significant differences by gender and age. At the same time there was a significant association between arsenic exposure and socio-economic status, with a higher percentage of socio-economically disadvantaged people in the group of those exposed to arsenic values > 10 µg/l (*p* < 0.0001).

### Associations between health outcomes and two-level As exposure

Table 4 shows the results of the associations between arsenic concentrations in drinking water > 10 µg/l and mortality/hospitalization outcomes, compared to the reference class (TWA ≤ 10 µg/l). We found higher risks of non-accidental mortality (HR = 1.07 95%CI: 1.01–1.13), more pronounced in women (HR = 1.10 95%CI: 1.02–1.19) than in men (HR = 1.04 95%CI: 0.95–1.12). Mortality for malignant cancer showed a positive association for women only (HR = 1.14 95%CI: 0.97–1.35). Long-term exposure to arsenic concentrations > 10 µg/l resulted positively associated with multiple hospitalization outcomes. Higher risks were observed for hospital admissions for non-accidental causes (HR = 1.06 95%CI: 1.03–1.09), both in women (HR = 1.07 95%CI: 1.03–1.12) and in men (HR = 1.05 95%CI: 1.01–1.10), for malignant neoplasms (HR = 1.10 95%CI: 1.02–1.19; women HR = 1.19 95%CI: 1.07–1.32), for lung cancer (HR = 1.85 95%CI: 1.14–3.02) and breast cancer (HR = 1.23 95%CI: 0.99–1.51) in women, for endocrine disorders (HR = 1.13 95%CI: 1.02–1.26; women HR = 1.17 95%CI: 1.02–1.35), for cardiovascular diseases (HR = 1.12 95%CI: 1.06–1.18; women HR = 1.13 95%CI: 1.05–1.22; men HR = 1.10 95%CI: 1.03–1.19), particularly for ischaemic heart disease (HR = 1.11 95%CI: 0.99–1.24), hypertensive disease (HR = 1.21 95%CI: 0.99–1.48), heart failure (HR = 1.14 95%CI: 1.01–1.29; women HR = 1.17 95%CI: 0.99–1.37), cerebrovascular diseases (HR = 1.07 95%CI: 0.98–1.17) and respiratory diseases (HR = 1.10 95%CI: 1.03–1.18; women HR = 1.11 95%CI: 1.01–1.23; men HR = 1.10 95%CI: 1.00–1.20).

**Table 4:** Associations between exposure to arsenic concentrations in drinking water > 10 µg/l and health outcomes

	**MORTALITY 1998–2016**						**HOSPITALIZATION 1998–2019**					
	WOMEN		MEN		TOTAL		WOMEN		MEN		TOTAL	
	Ref.^a^ N	TWA^b^ > 10 µg/l N HR (95%CI)^**c**^	Ref N	TWA > 10 µg/l N HR (95%CI)	Ref N	TWA > 10 µg/l N HR (95%CI)	Ref N	TWA > 10 µg/l N HR (95%CI)	Ref N	TWA > 10 µg/l N HR (95%CI)	Ref N	TWA > 10 µg/l N HR (95%CI)
Non-accidental causes	1367	1401 1.10 (1.02–1.19)	1401	1198 1.04 (0.95–1.12)	2768	2599 1.07 (1.01–1.13)	5530	4574 1.07 (1.03–1.12)	4811	3717 1.05 (1.01–1.10)	10,341	8291 1.06 (1.03–1.09)
Malignant neoplasms	309	304 1.14 (0.97–1.35)	501	421 1.00 (0.88–1.15)	819	725 1.06 (0.95–1.17)	747	700 1.19 (1.07–1.32)	936	749 1.03 (0.93–1.14)	1683	1449 1.10 (1.02–1.19)
Lung	25	28 1.42 (0.81–2.47)	119	109 1.08 (0.82–1.42)	144	137 1.14 (0.89–1.46)	32	43 1.85 (1.14–3.02)	145	125 1.05 (0.81–1.35)	177	168 1.18 (0.95–1.48)
Colon-rectum	51	39 0.87 (0.56–1.35)	64	50 0.91 (0.62–1.35)	115	89 0.90 (0.67–1.20)	102	92 1.14 (0.85–1.53)	143	99 0.89 0.67–1.16)	245	191 0.99 (0.81–1.21)
Kidney		n.d.^d^		n.d		n.d	25	21 0.99 (0.53–1.82)	48	30 0.87 (0.54–1.41)	73	51 0.91 (0.62–1.33)
Breast	36	31 1.03 (0.62–1.71)		n.d		n.d	199	191 1.23 (0.99–1.51)		n.d	0	n.d
Bladder	6	5 0.67 (0.19–2.39)	32	29 1.01 (0.59–1.72)	38	34 0.94 (0.58–1.55)	20	28 1.50 (0.81–2.77)	107	102 1.19 (0.89–1.58)	127	130 1.23 (0.95–1.60)
Stomach	32	42 1.51 (0.93–2.46)	56	41 0.93 (0.61–1.43)	88	83 1.14 (0.83–1.57)	51	52 1.16 (0.77–1.75)	88	66 0.98 (0.69–1.37)	139	118 1.05 (0.81–1.36)
Liver	23	20 0.99 (0.52–1.85)	53	27 0.69 (0.42–1.12)	76	47 0.78 (0.53–1.15)	33	26 0.96 (0.56–1.66)	76	47 0.81 (0.55–1.19)	109	73 0.86 (0.63–1.17)
Ovary	15	16 1.16 (0.55–2.45)		-		-	32	29 1.22 (0.72–2.07)		-	0	-
Prostate		-	37	39 1.19 (0.74–1.90)		-		-	119	112 1.27 (0.97–1.66)	0	-
Lymphatic and haematopoietic tissue	27	30 1.25 (0.73–2.14)	27	28 1.28 (0.73–2.25)	54	58 1.27 (0.86–1.88)	61	54 1.11 (0.75–1.63)	57	50 1.22 (0.81–1.84)	118	104 1.16 (0.88–1.54)
Endocrine disorders		n.d		n.d		n.d	473	460 1.17 (1.02–1.35)	336	275 1.08 (0.91–1.28)	809	735 1.13 (1.02–1.26)
Diabetes	42	42 1.01 (0.64–1.58)	30	25 0.96 (0.55–1.68)	72	67 0.99 (0.70–1.40)	132	142 1.13 (0.82–1.56)	132	100 0.96 (0.66–1.39)	264	242 1.03 (0.81–1.30)
Circulatory system	622	595 0.98 (0.87–1.10)	478	401 0.99 (0.86–1.14)	1100	996 0.99 (0.90–1.08)	1654	1570 1.13 (1.05–1.22)	1762	1490 1.10 (1.03–1.19)	3416	3060 1.12 (1.06–1.18)
Ischaemic heart disease	125	128 1.03 (0.79–1.34)	135	127 1.05 (0.81–1.36)	260	255 1.04 (0.87–1.25)	274	263 1.11 (0.93–1.33)	441	385 1.10 (0.95–1.27)	715	648 1.11 (0.99–1.24)
Acute myocardial infarction	52	58 1.20 (0.81–1.79)	64	57 0.98 (0.67–1.44)	116	115 1.08 (0.82–1.42)	161	156 1.13 (0.89–1.42)	261	206 0.98 (0.81–1.19)	422	362 1.04 (0.90–1.21)
Hypertensive disease		n.d		n.d		n.d	145	166 1.19 (0.90–1.56)	134	152 1.22 (0.90–1.65)	279	318 1.21 (0.99–1.48)
Heart failure	16	25 1.52 (0.79–2.92)	20	17 0.93 (0.46–1.84)	36	42 1.21 (0.75–1.93)	309	340 1.17 (0.99–1.37)	290	271 1.11 (0.93–1.32)	599	611 1.14 (1.01–1.29)
Cerebrovascular diseases	206	186 0.92 (0.75–1.13)	131	104 0.96 (0.73–1.25)	337	290 0.93 (0.79–1.10)	549	546 1.09 (0.96–1.24)	535	453 1.05 (0.92–1.20)	1084	999 1.07 (0.98–1.17)
Respiratory system	85	78 0.94 (0.68–1.30)	150	126 1.05 (0.81–1.34)	235	204 1.01 (0.83–1.23)	937	837 1.11 (1.01–1.23)	1111	924 1.10 (1.00–1.20)	2048	1761 1.10 (1.03–1.18)
COPD^e^	27	30 1.20 (0.70–2.08)	55	52 1.07 (0.71–1.60)	82	82 1.13 (0.81–1.56)	128	135 1.23 (0.95–1.60)	191	164 1.03 (0.83–1.30)	319	299 1.12 (0.94–1.32)
Urinary system	23	23 0.95 (0.51–1.77)	29	25 1.02 (0.58–1.77)	52	48 0.98 (0.65–1.48)	364	333 1.10 (0.94–1.29)	501	378 0.99 (0.86–1.14)	865	711 1.04 (0.93–1.15)
Chronic kidney disease	20	23 1.09 (0.57–2.07)	25	24 1.11 (0.62–1.99)	45	47 1.09 (0.71–1.68)	98	121 1.28 (0.96–1.70)	150	117 0.93 (0.72–1.21)	248	238 1.07 (0.89–1.30)
Diseases of the skin		n.d		n.d		n.d	207	148 0.92 (0.73–1.16)	257	207 1.13 (0.93–1.38)	464	355 1.04 (0.89–1.20)

^a^*Ref* Reference class TWA ≤ 10 µg/l

^b^*TWA* time-weighted average arsenic concentration in drinking water 2005–2010

^**c**^*HR* Hazard ratios from Cox proportional hazard models, adjusted for gender, socio-economic status and calendar period

^d^*n.d.* Not determined

^e^*COPD* Chronic obstructive pulmonary disease

### Associations between health outcomes and three-level As exposure

Table 5 shows the HR and 95% confidence intervals referring to associations between mortality/hospitalization outcomes and exposure to arsenic ​​in drinking water, using the TWA metric as a three-level categorical variable. Arsenic concentrations < 5 µg/l were selected as the reference class and two increasing exposure groups were considered: exposure below the regulatory standard (5–10 µg/l) and above (> 10 µg/l). The excess for non-accidental mortality was confirmed in the highest exposure group (HR 1.13 95%CI: 1.02–1.26) and a significant trend was observed (*p* = 0.009). Several associations were also highlighted in hospital admissions, in relation to the exposure group 5–10 µg/l, though the division into three groups generates a greater instability of the estimates. Compared to models with two-level TWA metric, the excess risks associated with the highest exposure class are mostly confirmed, such as hospitalizations for non-accidental causes, for endocrine disorders and cardiovascular diseases. However, results also showed increased risks for the intermediate exposure group (5–10 µg/l) and significant exposure related trend. This is the case for hospitalizations for ischaemic heart disease (5–10 µg/l class HR = 1.26 95%CI: 1.00–1.59), prostate cancer (*p* = 0.041), endocrine disorders (*p* = 0.015), cardiovascular diseases (*p* < 0.001), heart failure (*p* = 0.019) and respiratory diseases (*p* = 0.014).

**Table 5:** Associations between exposure to arsenic concentrations in drinking water below the regulatory limit (5–10 µg/l) and health outcomes

	**MORTALITY 1998–2016**						**HOSPITALIZATION 1998–2019**					
	**Ref.**^**a**^	**TWA**^**b**^ **5–10 µg/l**		**TWA** ** > 10 µg/l**		***p*-value trend**	**Ref**	**TWA** **5–10 µg/l**		**TWA** ** > 10 µg/l**		***p*-value trend**
	**N**	**N**	**HR (95%CI)**^**c**^	**N**	**HR (95%CI)**		**N**	**N**	**HR (95%CI)**	**N**	**HR (95%CI)**	
Non-accidental causes	400	2368	1.07 (0.96–1.20)	2599	1.13 (1.02–1.26)	0.009	1362	8979	1.03 (0.97–1.09)	8291	1.09 (1.03–1.15)	< 0.001
Malignant neoplasms	130	689	0.90 (0.74–1.10)	725	0.97 (0.80–1.17)	0.604	253	1430	0.89 (0.78–1.03)	1449	1.01 (0.88–1.15)	0.094
Lung	32	112	*0.57* *(0.38–0.86)*	137	0.74 (0.50–1.09)	0.930	33	144	0.70 (0.47–1.03)	168	0.89 (0.61–1.29)	0.524
Colon-rectum	22	93	0.77 (0.47–1.28)	89	0.73 (0.45–1.18)	0.274	43	202	0.72 (0.51–1.02)	191	0.76 (0.55–1.07)	0.413
Breast	5	31	0.99 (0.37–2.67)	31	1.02 (0.39–2.64)	0.932	26	173	1.01 (0.66–1.54)	191	1.23 (0.82–1.86)	0.082
Bladder	11	27	0.56 (0.26–1.24)	34	0.63 (0.31–1.29)	0.430	26	101	0.69 (0.44–1.10)	130	0.93 (0.60–1.43)	0.413
Stomach	10	78	1.34 (0.68–2.67)	83	1.46 (0.76–2.82)	0.276	19	120	1.12 (0.67–1.87)	118	1.15 (0.70–1.89)	0.626
Liver	9	67	1.19 (0.57–2.47)	47	0.90 (0.44–1.85)	0.346	13	96	1.16 (0.64–2.11)	73	0.97 (0.54–1.75)	0.494
Ovary	< 3	n.d. ^d^	n.d	n.d	n.d	n.d	5	27	0.73 (0.28–1.95)	29	0.94 (0.36–2.44)	0.665
Prostate	< 3	n.d	n.d	n.d	n.d	n.d	12	107	1.47 (0.80–2.72)	112	1.76 (0.97–3.19)	0.041
Lymphatic and haematopoietic tissue	4	50	2.16 (0.76–6.13)	58	2.47 (0.89–6.82)	0.097	16	102	0.97 (0.56–1.67)	104	1.13 (0.67–1.92)	0.369
Endocrine disorders	11	87	1.46 (076–2.81)	89	1.38 (0.774–2.60)	0.583	99	710	1.11 (0.89–1.38)	735	1.23 (1.00–1.53)	0.015
Diabetes	9	63	1.39 (0.67–2.90)	67	1.29 (0.64–2.61)	0.761	32	232	1.07 (0.65–1.74)	242	1.09 (0.67–1.76)	0.764
Circulatory system	156	944	1.17 (0.98–1.41)	996	1.12 (0.94–1.33)	0.683	483	2933	1.03 (0.93–1.13)	3060	1.14 (1.04–1.26)	< 0.001
Ischaemic heart disease	42	218	1.06 (0.74–1.50)	255	1.09 (0.78–1.51)	0.608	88	627	1.26 (1.00–1.59)	648	1.34 (1.07–1.67)	0.018
Acute myocardial infarction	18	98	1.07 (0.63–1.83)	115	1.14 (0.69–1.89)	0.537	53	369	1.21 (0.90–1.64)	362	1.22 (0.91–1.63)	0.331
Heart failure	4	32	1.78 (0.60–5.27)	42	1.93 (0.69–5.40)	0.264	87	512	1.13 (0.89–1.43)	611	1.26 (1.00–1.57)	0.019
Cerebrovascular diseases	53	284	1.00 (0.73–1.37)	290	0.93 (0.69–5.40)	0.441	170	914	0.96 (0.81–1.14)	999	1.04 (0.88–1.22)	0.250
Respiratory system	30	205	1.35 (0.89–2.05)	204	1.29 (0.87–1.92)	0.509	284	1764	0.97 (0.85–1.11)	1761	1.08 (0.95–1.22)	0.014
COPD^e^	10	72	1.64 (0.79–3.39)	82	1.68 (0.84–3.36)	0.242	41	278	1.26 (0.89–1.77)	299	1.34 (0.97–1.86)	0.094
Urinary system	5	47	2.09 (0.80–5.49)	48	1.79 (0.71–4.52)	0.601	113	752	1.09 (0.89–1.34)	711	1.11 (0.91–1.36)	0.356
Chronic kidney disease	4	41	2.41 (0.83–7.03)	47	2.24 (0.80–6.25)	0.312	35	213	1.24 (0.85–1.81)	238	1.27 (0.88–1.84)	0.270
Diseases of the skin	< 3	n.d	n.d	n.d	n.d	n.d	54	410	1.06 (0.79–1.43)	355	1.09 (0.81–1.46)	0.563

^a^*Ref* Reference class TWA ≤ 5 µg/l

^b^*TWA* Time-weighted average arsenic concentration in drinking water 2005–2010

^**c**^*HR* Hazard ratios from Cox proportional hazard models, adjusted for gender, socio-economic status and calendar period

^d^*n.d.* not determined

^e^*COPD* chronic obstructive pulmonary disease

## Discussion

This residential cohort study in the Mt. Amiata area found that chronic exposure to arsenic concentrations in drinking water above the current regulatory limit is associated with a plurality of diseases. Whilst some of these associations were observed in both men and women, women showed increased risks for several outcomes, in the analyses of both mortality and hospitalization. Positive associations were reported for non-accidental causes, malignant neoplasms, endocrine disorders, cardiovascular, and respiratory diseases. Moreover, our study also revealed some signs of associations even at very low exposure levels, below the current regulatory limit, in subjects expos​​ed to long-term arsenic concentrations in the range 5–10 µg/l. Significant exposure-related trends were observed for ischaemic heart disease, prostate cancer, endocrine disorders, cardiovascular diseases, heart failure and respiratory diseases. However, there was high uncertainty of these estimates due to the low number of cases, mainly in the reference class of subjects exposed to arsenic concentrations < 5 µg/l.

We found a positive association between arsenic exposure and lung cancer in females. Conflicting results have been obtained in studies of arsenic and cancer conducted in non-endemic areas [38–42]. Many studies focus on the effects on lung and bladder cancer and the findings reported are inconsistent [14, 15, 43–45]. Considering the available evidence on biological plausibility, our findings are coherent with the IARC evaluation regarding the carcinogenic role of arsenic on lung cancer [11]. We also find a positive association with breast cancer, for which there is limited epidemiological evidence in both high and low dose studies [11].

Our results also showed a consistent association with cardiovascular diseases in both genders, specifically for ischaemic heart diseases, hypertension, heart failure and cerebrovascular diseases. These findings are supported by a series of recent surveys on populations exposed to low or moderate arsenic concentrations, which have shown preclinical heart damage, such as increased mean intimal thickness, carotid plaques, endothelial dysfunction and vascular inflammation [9, 46].

In addition, we found consistent associations between arsenic exposure and respiratory diseases in both genders. For chronic obstructive pulmonary disease (COPD), women showed a higher risk than men. Our findings are supported by many international studies that have analysed the effects of arsenic on symptoms and respiratory function [17]. Some possible mechanisms of action have been formulated, such as the accumulation of arsenic in the pulmonary epithelium [17] or early epithelial changes detected by serum Clara cell protein CC16 concentrations [47]. All these findings, including those from our population-based cohort study in the Mt. Amiata area, significantly contribute to the debate on lowering the limit for arsenic concentrations in drinking water [48, 49].

Our study has several important strengths. First, the study benefited from the considerable size of the investigated population, which enabled the evaluation of associations with various mortality and hospitalization outcomes. Second, the longitudinal study design allowed us to assess indidually the time at risk for each resident in the study region. The reconstruction of the open and dynamic historical cohort was very accurate, and all residential movements were taken into account, including intra-municipality changes of address. Links to regional health administrative data allowed us to follow up on the study participants continuously and very effectively, reducing the risk of selection, recall or non-response bias. The long follow-up period allowed us to evaluate the arsenic exposure effects for long-latency pathologies, such as cancer. The use of GIS technology is in line with other international studies [14, 50, 51] and it enabled us to estimate the long-term exposure to arsenic in drinking water on an individual level, considering the time lived at each address. Based on a complete and accurate regional geographical database, the geocoding process provided high-quality and complete geocoded addresses, limiting the risk of exposure misclassification. In the cohort study in Amiata a time-weighted average of arsenic levels in drinking water was derived from data collected by the water supplier over the period 2005–2010. Before 2005, the only data available were those collected by the Local Health Authority but, for reasons of poor accuracy and comparability, we chose not to use them in the exposure assessment. However, available data assume that arsenic concentrations were constant before 2005.

There are some limitations to our research. Our study is population-based and we used administrative health databases with routinely collected data. This approach means that data on other potential individual risk factors, such as consumption of tobacco and alcohol, physical activity, diet and obesity, were not available and results could involve confounding bias. However, many personal habits are associated with socio-economic status; we applied the social deprivation index to adjust in our models which could have been limited bias due to unmeasured confounders. Nevertheless, having used an aggregated socioeconomic indicator at the census tract level does not eliminate the risk of ecological fallacy that may have led to misclassification on an individual level. Our exposure assessment only considered arsenic levels in drinking water, and we cannot exclude the fact that the real population exposure may have been underestimated, due to all potential sources other than drinking water. The potential role of the use of water from non-controlled private wells or the ingestion of contaminated food, such as locally produced vegetables or fruits, were not considered. Real arsenic intake is not known and is very difficult to quantify; it has been estimated that 35–45% of inorganic arsenic exposure in populations residing in non-endemic areas is attributable to the consumption of drinking water, 30% to the use of water to prepare food and the remaining 25–30% to food intake [52]. Regarding other potential sources of environmental exposure, in the same study area we previously investigated the role of long-term exposure to emissions from geothermal power plants, especially hydrogen sulphide, using the same population-based cohort [27]. Results indicated no associations between increased hydrogen sulphide levels and mortality/hospitalization outcomes, except for respiratory diseases. We considered both environmental exposures, arsenic in drinking water and atmospheric hydrogen sulphide in sensitivity analyses. In bi-pollutant models, associations with long-term exposure to arsenic in drinking water persisted for all outcomes.

To conclude, our findings from the population-based cohort study in the volcanic area of Mt. Amiata reinforce the existing evidence of the adverse effects of long-term exposure to low-level arsenic in drinking water. These results contribute to the current debate on the dose–response model and the need for a more protective threshold for arsenic risk assessment. However, our study in an area of naturally occurring arsenic highlights the need for continuous monitoring of arsenic concentrations and for policies to reduce concentrations in the environment as far as possible.

## Data Availability

There are legal restrictions on sharing a de-identified data set because data contain potentially identifying and sensitive people information (age, gender, residence, health status). Aggregated data may be requested by writing to Daniela Nuvolone at: daniela.nuvolone@ars.toscana.it.

## Abbreviations

As: Arsenic
TWA: Time-weighted average
Hg: Mercury
HR: Hazard ratio
CI: Confidence interval
SD: Standard deviation
COPD: Chronic obstructive pulmonary disease
WHO: World Health Organization
IARC: International Agency for Research on Cancer
GIS: Geographical information system
